# Hematocrit control and thrombotic risk in patients with polycythemia vera treated with ruxolitinib in clinical practice

**DOI:** 10.1007/s00277-024-05735-7

**Published:** 2024-04-25

**Authors:** Aleksander Chojecki, Danielle Boselli, Allison Dortilus, Issam Hamadeh, Stephanie Begley, Tommy Chen, Rupali Bose, Nikolai Podoltsev, Amer M. Zeidan, Nicole Baranda Balmaceda, Abdulraheem Yacoub, Jing Ai, Thomas Gregory Knight, Brittany Knick Ragon, Nilay Arvind Shah, Srinivasa Reddy Sanikommu, James Symanowski, Ruben Mesa, Michael Richard Grunwald

**Affiliations:** 1 Department of Hematologic Oncology and Blood Disorders, Atrium Health Levine Cancer Institute, Wake Forest University School of Medicine, Charlotte, NC USA; 2 Department of Biostatistics and Data Sciences, Levine Cancer Institute, Atrium Health, Charlotte, NC USA; 3 Department of Biostatistics, Memorial Sloan Kettering Cancer Center, New York, NY USA; 4 Hematology Section, Department of Medicine, Yale University School of Medicine, New Haven, CT USA; 5 Department of Hematologic Malignancies and Cellular Therapies, Kansas University, Kansas City, KS USA

**Keywords:** Polycythemia vera, Ruxolitinib, Retrospective studies, Hematocrit, Thrombosis, Myeloproliferative disorders

## Abstract

Polycythemia vera (PV) is a myeloproliferative neoplasm characterized by unregulated red blood cell production resulting in elevated hemoglobin and/or hematocrit levels. Patients often have symptoms such as fatigue, pruritus, and painful splenomegaly, but are also at risk of thrombosis, both venous and arterial. Ruxolitinib, a selective Janus kinase inhibitor, is approved by the US Food and Drug Administration as second-line cytoreductive treatment after intolerance or inadequate response to hydroxyurea. Although ruxolitinib has been widely used in this setting, limited data exist in the literature on ruxolitinib treatment patterns and outcomes among patients with PV in routine clinical practice. We report a retrospective, observational, cohort study of patients treated for PV with ruxolitinib across three US centers (academic and regional practice) from December 2014-December 2019. The study included 69 patients, with a median follow-up duration of 3.7 years (95% CI, 2.9–4.4). Our data demonstrate very high rates of hematocrit control (88% of patients by three months and 89% by six months); few patients required dose adjustments or suspension. No arterial thromboses were observed; however, the follow-up duration does not allow for the generation of meaningful conclusions from this. Three patients had thrombotic events; one was in the setting of a second malignancy, one post-operative, and a third related to prolonged immobility. We also found that 28% of patients initiated ruxolitinib as a result of poorly controlled platelet counts, second only to hydroxyurea intolerance (46%) as a reason to start therapy. In clinical practice, ruxolitinib continues to be effective in controlling hematocrit levels after three and six months of treatment in patients and is associated with low thrombotic risk.

## Main text

Polycythemia vera (PV) is a myeloproliferative neoplasm characterized by unregulated red blood cell production. Elevated hemoglobin (HGB) and/or hematocrit (HCT) are required for PV diagnosis [1]. Clinical symptoms of PV that can impact quality of life include fatigue, pruritus, and painful splenomegaly [2]. Moreover, patients with PV are also at increased risk of thrombosis, both venous and arterial [3, 4]. Investigators of a large multi-center study of 1545 patients reported arterial thrombosis in 16% of patients and venous thrombosis in 7%, either at or before diagnosis [5]. A conventional risk stratification system is used to guide treatment selection according to age and/or history of a thrombotic event [6]. Patients at high risk are those aged 60 or older and/or with a history of thrombotic events; otherwise, patients are considered low risk. Low risk patients are typically treated with aspirin and therapeutic phlebotomy to achieve HCT control (HCT < 45%). Along with these measures, high risk patients can be recommended cytoreductive therapies such as hydroxyurea and interferons. In 2014, on the basis of the phase 3 RESPONSE trial results, the US Food and Drug Administration approved ruxolitinib, a selective Janus kinase (JAK) inhibitor, as second-line cytoreductive treatment after intolerance or inadequate response to hydroxyurea [7]. Five-year results showed ruxolitinib to be a safe and effective long-term treatment option for PV [8]. Thromboembolic events were lower in the ruxolitinib group than in the best-available therapy group.

Limited data exist in the literature on ruxolitinib treatment patterns and outcomes among patients with PV in routine clinical practice. Results of a recent US multi-center retrospective analysis showed that patients who received ruxolitinib had durable HCT control and decreased need for therapeutic phlebotomy [9]. Additionally, results from a real-world setting in Spain suggested decreased rates of arterial thrombotic events with ruxolitinib [10]. A US-based community chart review showed that ruxolitinib treatment at manufacturer-recommended dosing (10 mg twice daily) correlated with safety and efficacy outcomes [11].

We conducted a retrospective, observational, cohort study of patients treated for PV with ruxolitinib at one of three US centers – Yale University (*n* = 9), Kansas University (*n* = 12), and Levine Cancer Institute (*n* = 30 at tertiary hub in Charlotte, North Carolina; *n* = 18 at regional sites) – from December 2014-December 2019. Our objective was to evaluate patterns of care and outcomes with ruxolitinib use in patients diagnosed with PV. Exclusion criteria included age < 18 at initiation of ruxolitinib, diagnosis of myelofibrosis, and receipt of ruxolitinib for PV via an investigational study. This study was designed according to Good Clinical Practice guidelines and the Declaration of Helsinki and was approved by local institutional review boards.

From the electronic medical records (EMR), we collected data related to PV diagnosis, demographic information, and PV-associated treatments and outcomes including changes in HCT levels and response, phlebotomy requirements, and thrombotic events. We also collected data on the reasons ruxolitinib was used, non-hematological side effects, dose adjustments/suspensions due to cytopenias, best response to ruxolitinib, and reasons for discontinuing ruxolitinib. We collected data for HCT levels (%) within one month (± 1 week) before ruxolitinib initiation (defined as baseline), three months (± 1 month) after initiating ruxolitinib, and six months (± 1 month) after initiating ruxolitinib. We summarized and described data for patient, disease, and treatment characteristics, and we computed follow-up length. We compared clinical outcomes before initiation of ruxolitinib to those at three and six months thereafter to evaluate rates of HCT control (HCT < 45%), number of phlebotomies (using a paired t-test), thrombotic events, and dose reductions/suspensions due to cytopenias. We collected information for side effects and reasons for therapy discontinuation throughout the study period of five years. All analyses were conducted in SAS System for Windows v9.4 (SAS Institute).

We identified 69 patients for inclusion (Table 1). Fifty-eight (84%) were deemed high risk and 11 (16%) low risk. PV data from the time of diagnosis were available for 62 patients; median age at diagnosis was 60.5 (24–95) years. Sixty-two of 69 patients (90%) received cytoreductive therapy before ruxolitinib, most commonly hydroxyurea alone (*n* = 47), but also anagrelide alone, pegylated interferon alone, or combination treatment. Seven patients received phlebotomy or aspirin/anticoagulation alone. The median time from PV diagnosis to initiating ruxolitinib was 4.4 (0.1–40.2) years. The most common reasons for initiating ruxolitinib were hydroxyurea intolerance (32 of 69; 46%), uncontrolled platelet count (19 of 69; 28%), and burdensome symptoms (13 of 69; 19%; Table 1). Less common reasons included splenomegaly (10 of 69; 14%), hydroxyurea non-responsiveness (9 of 69; 13%), poor tolerance of phlebotomies (4 of 69; 6%), and symptomatic iron deficiency (4 of 69; 6%).

**Table 1:** Characteristics of 69 patients with polycythemia vera and their treatment history before ruxolitinib treatment

Patient and disease characteristics	*N* (%)
*Site*	
Levine Cancer Institute (Atrium Health)	48 (70)
University of Kansas Medical Center	12 (17)
Yale Cancer Center	9 (13)
*Age at PV diagnosis (n = 62)*, Median [Range]	60.5 [24, 95]
*Sex*	
Female	32 (46)
Male	37 (54)
*Race*	
Black or African American	4 (6)
White	59 (85)
Other	4 (6)
Unknown	2 (3)
*Ethnicity*	
Hispanic or Latino	3 (4)
Non-Hispanic or Latino	54 (93)
Unknown	2 (3)
*Risk*	
Low	11 (16)
High	58 (84)
*Prior therapies*	
Hydroxyurea only ± phlebotomy	47 (68)
Combination cytoreduction ± phlebotomy	12 (18)
Phlebotomy only (no cytoreduction)	6 (9)
Anagrelide only ± phlebotomy	2 (3)
Aspirin, anticoagulants (non-PV therapy) only	1 (1)
Pegylated interferon only ± phlebotomy	1 (1)
*Reasons for initiating ruxolitinib* ^*a*^	
Hydroxyurea intolerance	32 (46)
Uncontrolled platelet count	19 (28)
Burdensome or intractable symptoms	13 (19)
Splenomegaly and/or spleen-associated symptoms	10 (14)
Hydroxyurea non-responsiveness	9 (13)
Uncontrolled hematocrit	7 (10)
Uncontrolled WBC	7 (10)
Intolerance to other therapies	6 (9)
Poor tolerance of phlebotomy	4 (6)
Symptomatic iron deficiency	4 (6)
Cytopenias interfering with adequate control of hematocrit	2 (3)
Non-responsiveness to other therapies	1 (1)
Others	7 (10)
*Reasons for discontinuing ruxolitinib* ^*a*^ *(n = 26)*	
Body aches	5 (19)
Fatigue	5 (19)
Cytopenias	4 (15)
Terminal illness	4 (15)
Infection	3 (12)
Dizziness	2 (8)
Self-discontinued	2 (8)
Nausea	2 (8)
Progression to AML or no response	2 (8)
Abdominal pain	1 (4)
Loss of financial coverage	1 (4)
Weight gain	1 (4)
Other	1 (4)

^*a*^ Some patients had more than one reason for initiating or discontinuing ruxolitinib

Median follow-up duration was 3.7 years (95% CI, 2.9 to 4.4). At the conclusion of the observation periods, 43 patients (62%) were on ruxolitinib; the Kaplan-Meier estimate of median time on treatment was 5.3 years (95% CI, 4.4 to non-estimable). Of the 26 patients who discontinued ruxolitinib (Table 1), eight patients (12%) were treated with ruxolitinib for less than six months. Reasons for discontinuation of therapy within 6 months included fatigue (*n* = 4), body aches (*n* = 3), dizziness (*n* = 2), nausea (*n* = 2), weight gain (*n* = 1), cytopenias (*n* = 1), abdominal pain (*n* = 1), and intolerance (*n* = 1); most patients had more than one reason for discontinuing. HCT control at baseline was observed for 59% of patients (Table 2). Three months after initiating ruxolitinib, the overall HCT control rate was 88%; 91% in patients with HCT control at ruxolitinib initiation and 83% in those without HCT control at baseline. Six months after initiating ruxolitinib, the overall HCT control rate was 89%; 94% in patients with HCT control at ruxolitinib initiation and 82% in those without HCT control at baseline. We investigated the distributions of HCT response among the various pre-ruxolitinib therapeutic groups and did not detect differential outcomes (Table 2). At 3 months, durable peripheral hematologic remission – HCT < 45% without phlebotomy, platelet count < 400 × 10^9^/L, and WBC count < 10 × 10^9^/L – was observed in 44% and resolution of disease-related symptoms was observed in 83% of patients for whom data were available. Forty-seven patients (68%) reported non-hematologic side effects on ruxolitinib; the most common were weight gain (*n* = 10), dizziness (*n* = 9), headaches (*n* = 7), and nausea (*n* = 6), dyspnea (*n* = 5), edema (*n* = 3), and fatigue (*n* = 3).

**Table 2:** Hematocrit levels of patients with polycythemia vera before (baseline) and after initiation of ruxolitinib. HCT < 45% is classified as HCT control; HCT ≥ 45% as HCT not under control

**All patients**	**HCT < 45%**, ***n*(%)**	**HCT ≥ 45%**, ***n(%)***	**Total patients**
1 month^a^ before initiating ruxolitinib	39 (59)	27 (41)	*n* = 66
3 months^b^ after initiating ruxolitinib	53 (88)	7 (12)	*n* = 60
HCT < 45% at baseline	32 (91)	3 (9)	*n* = 35
HCT ≥ 45% at baseline	20 (83)	4 (17)	*n* = 24
6 months^c^ after initiating ruxolitinib	49 (89)	6 (11)	*n* = 55
HCT < 45% at baseline	31 (94)	2 (6)	*n* = 33
HCT ≥ 45% at baseline	18 (82)	4 (18)	*n* = 22
**Treatment groups**			
**Cytoreduction: Hydroxyurea only**	**HCT < 45%**, ***n*(%)**	**HCT ≥ 45%**, ***n*(%)**	**Total patients**
1 month^a^ before initiating ruxolitinib	26 (59)	18 (41)	*n* = 44
3 months^b^ after initiating ruxolitinib	34 (85)	6 (15)	*n* = 40
HCT < 45% at baseline	21 (91)	2 (9)	*n* = 23
HCT ≥ 45% at baseline	12 (75)	4 (25)	*n* = 16
6 months^c^ after initiating ruxolitinib	30 (83)	6 (17)	*n* = 36
HCT < 45% at baseline	19 (90)	2 (10)	*n* = 21
HCT ≥ 45% at baseline	11 (73)	4 (27)	*n* = 15
**Cytoreduction: Other** ^**d**^	**HCT < 45%**, ***n*(%)**	**HCT ≥ 45%**, ***n*(%)**	**Total patients**
1 month^a^ before initiating ruxolitinib	9 (60)	6 (40)	*n* = 15
3 months^b^ after initiating ruxolitinib	12 (92)	1 (8)	*n* = 13
HCT < 45% at baseline	7 (88)	1 (12)	*n* = 8
HCT ≥ 45% at baseline	5 (100)	0 (0)	*n* = 5
6 months^c^ after initiating ruxolitinib	12 (100)	0 (0)	*n* = 12
HCT < 45% at baseline	8 (100)	0 (0)	*n* = 8
HCT ≥ 45% at baseline	4 (100)	0 (0)	*n* = 4
**No cytoreduction**	**HCT < 45%**, ***n*(%)**	**HCT ≥ 45%**, ***n*(%)**	**Total patients**
1 month^a^ before initiating ruxolitinib	4 (57)	3 (43)	*n* = 7
3 months^b^ after initiating ruxolitinib	7 (100)	0 (0)	*n* = 7
HCT < 45% at baseline	4 (100)	0 (0)	*n* = 4
HCT ≥ 45% at baseline	3 (100)	0 (0)	*n* = 3
6 months^c^ after initiating ruxolitinib	7 (100)	0 (0)	*n* = 7
HCT < 45% at baseline	4 (100)	0 (0)	*n* = 4
HCT ≥ 45% at baseline	3 (100)	0 (0)	*n* = 3

^a^ One month ± 7 days; ^b^ 3 months ± 1 month; ^c^ 6 months ± 1 month; ^d^ includes anagrelide, pegylated interferon, or combination cytoreduction (hydroxyurea with at least one of the following pegylated interferon or anagrelide)

We assessed the association between ruxolitinib treatment and frequency of phlebotomies. Phlebotomy data for the three months before and after initiating ruxolitinib were available for 48 patients. After initiating ruxolitinib, 21 (44%) had fewer phlebotomies (-1 to -15), 20 (42%) remained stable, and 7 (14%) had more phlebotomies (+ 1 to + 3) (*P* = .013). Twelve patients (17%) required ruxolitinib dose reductions/suspensions due to cytopenias. Of these patients, seven (10%) were able to resume ruxolitinib. No arterial thromboses were observed; however, the follow-up duration was relatively short. Three patients experienced thrombotic events while on ruxolitinib. One patient experienced an acute pulmonary embolism more than four years after ruxolitinib initiation. This patient had been diagnosed with T-cell lymphoma and the thrombotic event was attributed to the lymphoma. It is unclear whether ruxolitinib use was related to the development of T-cell lymphoma. Another patient experienced an acute pulmonary embolism and bilateral lower extremity thrombosis more than three years after ruxolitinib initiation. This patient underwent resection of an anaplastic meningioma and was hospitalized 12 days later with hemorrhage and thrombosis, attributed to post-operative complications. The meningioma had been discovered before PV was diagnosed. A third patient experienced acute bilateral pulmonary embolisms four months after ruxolitinib initiation. This event was discovered shortly after a period of prolonged immobility during travel. All three of these patients had achieved HCT control on ruxolitinib at three and six months.

Several recent reports describe the association of ruxolitinib use with clinical efficacy in patients with PV in real-world settings [9–11]. Altomare and colleagues conducted a medical chart review of 249 patients and found that about half of patients (53%) were started on the standard recommended dose of 10 mg ruxolitinib twice daily, and 27% of these patients had dose modifications [11]. Most patients (63%) achieved HCT control within six months of initiating ruxolitinib. The RESPONSE trial reported HCT control of 60% at 32 weeks and RESPONSE-2 reported HCT control of 62% at 28 weeks [7, 12]. Our data demonstrate very high rates of HCT control (88% of patients by three months and 89% by six months), suggesting that not only is treatment effective but also patients in clinical practice can remain on ruxolitinib; few patients required dose adjustments or suspension during the study period. Pepe and colleagues reported a significant reduction in HCT levels and phlebotomy requirements in a cohort of 83 patients in Italy [13]. Our data corroborate these findings by demonstrating that most patients achieved HCT control by three months and many patients had decreased phlebotomy needs after initiating ruxolitinib. Results of a retrospective study conducted in Spain showed that ruxolitinib treatment was associated with fewer thrombotic events [10]. We found that three patients had thrombotic events, one in the setting of a second malignancy, one from post-operative complications, and a third correlated with prolonged immobility. Tremblay and colleagues performed a retrospective multi-institutional review of PV patients who discontinued ruxolitinib and found that adverse events were the most common reason for discontinuation [14]. In our study, 26 patients (38%) discontinued ruxolitinib, with most common reasons being fatigue (19%), body aches (19%), cytopenias (15%) and other terminal illness (15%). Lastly, we found that 28% of patients initiated ruxolitinib as a result of poorly controlled platelet counts, second only to hydroxyurea intolerance (46%). A high platelet count is not a widely recognized reason for changing therapy, and this finding may merit further exploration.

Our results complement those of other clinical studies; within three months of initiating ruxolitinib, most patients had HCT control and decreased phlebotomy needs. Although 68% of patients reported non-hematologic side effects, the rate of ruxolitinib discontinuation was low. For patients who discontinue ruxolitinib, other treatment strategies can be considered such as hydroxyurea or interferon therapy. Novel agents such as rusfertide are in development and show promising activity [15]. Our data also suggest that most patients achieved HCT control regardless of prior PV treatment and few developed thrombosis. Our study has several limitations. Firstly, we had a relatively small sample size and a short follow-up period that does not allow for the generation of meaningful conclusions from our observations of thrombosis events, for example. Secondly, many data elements were manually abstracted from the EMR and were subject to recall bias and/or were missing. Additionally, we did not collect detailed data on non-hematologic side effects, depth of cytopenias, dosing, level of dose reduction, or patients’ individual costs. Future studies could include longer follow-up and capture more details on side effects, dosing strategies, thrombotic risks, and ruxolitinib costs to patients.

Ruxolitinib remains a highly effective treatment option for patients with PV. In clinical practice, ruxolitinib continues to be effective in controlling HCT after three and six months of treatment in patients and is associated with low thrombotic risk.

## Data Availability

No datasets were generated or analysed during the current study.
